# Thermal Stability of Botulinum Toxin Type A Formulations Exposed to Heat In Vitro, Assessed by an In Vivo Mouse Potency Bioassay

**DOI:** 10.1093/asjof/ojag025

**Published:** 2026-02-06

**Authors:** Hyoung Moon Kim, Seongsung Kwak, Yu Sun Choi, Dongkyu Lee, Jin-Hee Kwon, Hyosan Jung

## Abstract

**Background:**

Energy-based devices (EBDs), such as high-intensity focused ultrasound, radiofrequency, and microwave, are increasingly used for facial rejuvenation, raising concerns about whether heat exposure may affect the potency of botulinum toxin.

**Objectives:**

This study investigated the thermal stability of 4 commercial botulinum toxin type A (BoNT/A) products under conditions simulating EBD-related heat exposure, which includes 1 liquid formulation (INNOTOX, Medytox, Seoul, South Korea; hereafter referred to as innoBoNT/A) and 3 powder formulations: onabotulinumtoxinA (onaBoNT/A, BOTOX, Allergan, an Abbvie company, North Chicago, IL), incobotulinumtoxinA (incoBoNT/A, XEOMIN, Merz, Frankfurt, Germany), and abobotulinumtoxinA (aboBoNT/A, DYSPORT, Ipsen, Boulogne-Billancourt, France).

**Methods:**

Mouse intraperitoneal LD_50_ potency assays were performed after exposing reconstituted and liquid formulations to 60°C for 10 to 40 min. Relative and normalized potency values were compared before and after exposure.

**Results:**

InnoBoNT/A maintained its potency after 25 min of exposure at 60°C, showing no significant loss of biological activity. In contrast, onaBoNT/A, incoBoNT/A, and aboBoNT/A displayed marked reduction in potency under the same conditions. In particular, onaBoNT/A showed a 32% decrease upon exposure at 60°C for 25 min and a complete loss of measurable potency at when exposed at 60°C for 40 min.

**Conclusions:**

Among the BoNT/A formulations evaluated, only the liquid-stabilized preparation preserved potency during thermal stress.

**Level of Evidence: 4 (Therapeutic):**

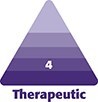

In recent years, the use of energy-based devices (EBDs) for facial rejuvenation has been increasing. For facial rejuvenation and other cosmetic purposes, commonly used EBDs include high-intensity focused ultrasound (HIFU), radiofrequency (RF) devices, intense pulsed light, and microwave-based devices.^1^ In the past, pretreatment with botulinum toxin before laser procedures such as periorbital resurfacing was a safe and effective method for reducing periorbital wrinkles.^2-4^ These findings have encouraged the combined use of botulinum toxin injections with various dermatologic procedures.

More recently, EBD treatment modalities have shifted toward long-pulse delivery and higher accumulated total energy for rejuvenation or skin tightening. In particular, the efficacy of newer devices is achieved not only by delivering higher energy per shot but also by prolonging treatment duration to accumulate sufficient energy.^5,6^ Such approaches can increase subdermal tissue temperatures up to 60°C or higher, depending on device type and treatment parameters.^7^ Along with this trend, there has been growing interest in whether the potency of botulinum toxin can get compromised following subsequent EBD treatment. It has become necessary to evaluate the effect of heat, comparable to that generated by the EBD procedure, on botulinum toxin potency.

Botulinum neurotoxins are protein complexes whose pharmacological effects manifest within a specific period of time after injection.^8,9^ However, its sensitivity and stability under physical stress can vary depending on the formulation and excipients used. Traditional botulinum toxin type A (BoNT/A) formulations (eg, onabotulinumtoxinA [BOTOX; Allergan, an Abbvie company, North Chicago, IL], incobotulinumtoxinA [XEOMIN; Merz, Frankfurt, Germany], abobotulinumtoxinA [DYSPORT; Ipsen, Boulogne-Billancourt, France]) contain human serum albumin as a stabilizer. They are provided as lyophilized powders, which may be susceptible to denaturation during reconstitution and thermal stress.^10,11^ In contrast, INNOTOX (Medytox, Seoul, South Korea; hereafter referred to as innoBoNT/A) is a ready-to-use BoNT/A liquid formulation that incorporates non-animal-derived stabilizers such as methionine and polysorbate 20, which have been suggested to improve protein stability against oxidative and thermal stress.^12-14^

If temperature fluctuations induced by EBDs influence the potency of BoNT/A, the treatment interval between toxin injection and EBD procedure should be carefully considered. In this study, different BoNT/A formulations and types (liquid vs lyophilized) were subjected to high temperature (60°C), simulating thermal conditions that may occur during EBD treatment, and the effects on toxin potency were evaluated. This study was conducted to provide experimental evidence and potential implications for the safe and effective integration of BoNT/A injections and EBD procedures in clinical practice.

## METHODS

### Commercial BoNT/A Products

Four commercially available BoNT/A formulations were investigated in this study: 1 liquid formulation (INNOTOX, hereafter referred to as innoBoNT/A) and 3 powder formulations (onabotulinumtoxinA [onaBoNT/A, BOTOX], incobotulinumtoxinA [incoBoNT/A, XEOMIN], and abobotulinumtoxinA [aboBoNT/A, DYSPORT]). A comprehensive summary of product-related information is described in Table 1.

**Table 1.** Characteristics of Commercially Available BoNT/A Products Used in the Study

Product	Manufacturer	Lot no.	Formulation	Active ingredient	Excipients	Labeled potency (U/vial)
InnoBoNT/A (INNOTOX)	Medytox	D124014	Liquid	900 kDa BoNT/A	Methionine, Polysorbate 20, NaCl	100
OnaBoNT/A (BOTOX)	Abbvie	D0204AC4	Powder	900 kDa BoNT/A	NaCl, HSA	100
IncoBoNT/A (XEOMIN)	Merz	435703	Powder	150 kDa BoNT/A	Sucrose, HSA	100
AboBoNT/A (DYSPORT)	Ipsen	010285	Powder	500-900 kDa BoNT/A complex^a^	Lactose, HSA	500

AboBoNT/A, abobotulinumtoxinA; BoNT/A, botulinum toxin type A; IncoBoNT/A, incobotulinumtoxinA; HSA, human serum albumin; OnaBoNT/A, onabotulinumtoxinA.

^a^Molecular weight not reported.

### Preparation of Reconstituted BoNT/A Products and Exposure to High Temperature

All BoNT/A products were prepared according to their labeled vial potencies: innoBoNT/A (100 U/vial), onaBoNT/A (100 U/vial), incoBoNT/A (100 U/vial), and aboBoNT/A (500 U/vial). The 3 BoNT/A powder formulations were first reconstituted with 2.5 mL saline per vial following manufacturer's instructions, after which each vial was sealed with parafilm. For the liquid formulation (innoBoNT/A), the vial was briefly opened to break the vacuum seal and then similarly sealed with parafilm. To simulate thermal exposure associated with EBD operations (up to ∼60°C), all 4 BoNT/A formulations were placed in a dry oven at 60°C for 10 to 40 min.^7,15,16^ The oven temperature was monitored throughout by confirming the displayed temperature during each run.

### Animals

Four-week-old female CD-1(ICR) mice were purchased from Orient Bio, Inc. (Seongnam-si, Republic of Korea), housed in groups of 5 in a 12 h light/dark cycle, and given free access to food and water. A total of 2520 mice were used in this study. All animal experiments were approved by the Institutional Animal Care and Use Committee of Medytox Inc. (approval no. A-2025-003; approval date April 2, 2025) in compliance with the Laboratory Animal Act of Korea (act no. 19918).

### Mouse Potency Assay

The mouse intraperitoneal median lethal dose (IP LD_50_) assay, or “mouse potency assay,” is currently the standard method for determining the potency of botulinum toxin products in South Korea. This method was adopted in the present study to assess the thermal stability of the botulinum toxin products in accordance with the Specifications and Test Methods for Biological Products issued by the Ministry of Food and Drug Safety (MFDS, 2024).^17^ Six serial dilutions of the BoNT/A preparations and an internal reference standard were prepared in saline starting at 35.7 U/mL. Each dilution of the BoNT/A preparation was administered intraperitoneally to 10 mice at a dose volume of 0.1 mL. The number of mice at each BoNT/A dilution that died within 72 h after IP administration of the BoNT/A preparation was recorded. The relative potency of each BoNT/A preparation was calculated using the quantal response parallel-line analysis method, and the normalized potency estimate was calculated by multiplying the relative potency by the nominal value of the internal reference standard.^18^ One mouse IP LD_50_ was defined as 1 unit (U). To comply with the 3Rs principles (replacement, reduction, and refinement) for conducting research involving animals, each experiment was conducted using a single set of dilutions alongside the internal reference standard. The result of the experiments was considered valid only when the normalized potency estimate of the BoNT/A preparation pre-exposure to high temperature is within 80% to 125% of the stated potency.^17,19^

### Data and Statistical Analyses

The IP LD_50_ analysis was conducted using CombiStats software (version 1.1.1, EDQM), following the method described in the mouse potency assay. Results were expressed as individual potency values. Statistical analysis was not performed for samples from the 3 replicate studies in which potency values could not be calculated at the 25 min time point, excluding innoBoNT/A.

## RESULTS

### Preliminary Potency Assessment of InnoBoNT/A and OnaBoNT/A to Determine Optimal Exposure Time at 60°C

**Figure 1.**
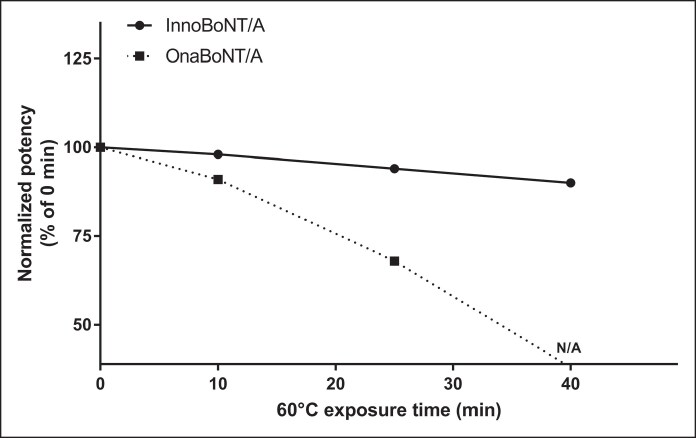
Thermal stability of innoBoNT/A and onaBoNT/A after exposure to 60°C. Normalized potency was calculated by setting the 0 min value as 100% and expressing subsequent results as percentage changes relative to baseline. N/A indicates no mortality was observed and potency was not measurable. BoNT/A, botulinum toxin type A.

To establish the optimal exposure time for subsequent thermal stability experiments, an initial potency assay was performed on innoBoNT/A and onaBoNT/A. The onaBoNT/A product was selected because it is the most widely used botulinum toxin preparation worldwide and, like innoBoNT/A, is derived from *Clostridium botulinum* type A-Hall strain. Normalized potency of the 2 BoNT/A formulations before (0 min), and after exposure to 60°C for 10, 25, and 40 min was measured. Potency of innoBoNT/A after exposure to 60°C for 40 min was only reduced by ∼10%. In contrast, the potency of onaBoNT/A was reduced to a level below the assay detection limit under the same thermal conditions because there were no animal deaths observed. When exposed to 60°C for only 25 min, the potency of innoBoNT/A decreased by only 6%, whereas the potency of onaBoNT/A was reduced by ∼32% (Figure 1, Table 2). Based on these findings, the 25 min exposure to 60°C was selected for comparing thermal stability.

**Table 2.** Potency Results According to Exposure Time at 60°C

Exposure time to 60°C (min)	InnoBoNT/A	OnaBoNT/A
	Relative potency^a^ (U/vial)	Relative potency^a^ (U/vial)
0	108	81
10	105	74
25	101	55
40	97	ND

BoNT/A, botulinum toxin type A; ND, not detected, no death of mice.

^a^Medytox internal reference standard was used.

### Comparative Thermal Stability of 4 Botulinum Toxin Preparations Following Exposure to 60°C

**Figure 2.**
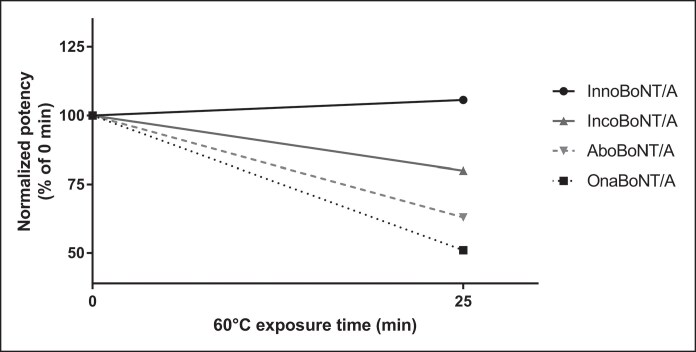
Thermal stability of innoBoNT/A, onaBoNT/A, incoBoNT/A, and aboBoNT/A after exposure to 60°C for 25 min. Potency values were normalized to the corresponding 0 min measurement (baseline = 100%). Data are presented as mean values, with excluded n/a results (no mortality observed, potency not measurable). Graphs were generated using only measurable replicate values. AboBoNT/A, abobotulinumtoxinA; BoNT/A, botulinum toxin type A; incoBoNT/A, incobotulinumtoxinA; onaBoNT/A, onabotulinumtoxinA.

Based on the in-house mouse potency assay using our internal reference standard, normalized potency estimates of 3 different batches of the BoNT/A preparations not exposed to high temperature (pre-exposure) were as follows: innoBoNT/A, 99 to 103 U/vial; onaBoNT/A, 83 to 92 U/vial; incoBoNT/A, 82 to 84 U/vial; and aboBoNT/A, 209 to 221 U/vial (Table 3). Following reconstitution in saline and exposure to 60°C for 25 min, all 3 batch preparations of innoBoNT/A maintained their potency (106-109 U/vial), demonstrating no loss of biological activity. Conversely, onaBoNT/A, incoBoNT/A, and aboBoNT/A, when reconstituted in saline, showed a significant loss in biological activity after exposure to 60°C for 25 min. Among the 3 mouse potency assays conducted for each product, potency could not be determined in 1 experiment for onaBoNT/A and in 2 experiments each for incoBoNT/A and aboBoNT/A because of the absence of mortality at the highest dose tested. These results suggest that the potency of these BoNT/A preparations was reduced to a level below the detection limit of the mouse potency assay. Two onaBoNT/A preparations showed potencies of 45 and 44 U/vial, indicating a 51% and 47% decrease, respectively, whereas the measured potencies of the remaining batch of incoBoNT/A (66 U/vial) and aboBoNT/A (140 U/vial) reflected a 20% and 37% decrease, respectively (Table 3, Figure 2). These findings strongly suggest that innoBoNT/A liquid botulinum toxin formulation exhibits superior thermal stability than the 3 powder BoNT/A formulations.

**Table 3.** Potency Results of 4 Botulinum Toxin Products After Exposure to 60°C for 25 min

BoNT/A formulations (labeled potency)	Potency (U/vial)^a^		Change in potency after exposure to 60°C, 25 min (%)^b^
	Pre-exposure	60°C, 25 min	
InnoBoNT/A (100 U/vial)	102	106	103
	99	106	108
	103	109	106
OnaBoNT/A (100 U/vial)	92	45	49
	85	ND	N/A
	83	44	53
IncoBoNT/A (100 U/vial)	84	ND	N/A
	82	ND	N/A
	82	66	80
AboBoNT/A (500 U/vial)	209	ND	N/A
	213	ND	N/A
	221	140	63

AboBoNT/A, abobotulinumtoxinA; BoNT/A, botulinum toxin type A; IncoBoNT/A, incobotulinumtoxinA; ND, not detected, no death of mice; N/A, not applicable; OnaBoNT/A, onabotulinumtoxinA.

^a^Normalized potency estimate using Medytox internal reference standard.

^b^Percentage relative to normalized potency estimate at pre-exposure.

## DISCUSSION

In this study, the mouse intraperitoneal LD_50_ assay was used to evaluate potency changes of different BoNT/A formulations under thermal stress. Variations between labeled and experimentally measured potencies likely arise from manufacturer-specific LD_50_ assay methodologies, which use distinct reference standards and assay conditions. As potency units are not interchangeable across commercial BoNT/A products, comparisons in this study should be interpreted within the context of these product-specific assay systems rather than assumed equivalence of labeled units.^20^

Cell-based potency assays for BoNT/A have already been developed and are increasingly recognized as suitable alternatives to animal testing.^21,22^ However, commercial BoNT/A drug products differ substantially in stabilizers and formulation matrices. As a consequence, differences in excipient compositions are likely to alter the constitution of the culture medium used for each BoNT/A formulation, thereby affecting the results of these assays. Hence, cell-based potency measurement was not considered appropriate for comparing thermal stability across different BoNT/A products.

Our findings demonstrated apparent formulation-dependent differences in thermal stability. The liquid formulation (innoBoNT/A) preserved biological potency after exposure to 60°C for up to 25 min, with no significant decline across replicates. In contrast, the powder-based preparations (onaBoNT/A, incoBoNT/A, and aboBoNT/A) exhibited substantial reductions in potency under the same conditions, with onaBoNT/A showing complete loss of measurable activity by 40 min. These results suggest that formulation type and excipients, rather than clostridial strain or neurotoxin molecular weight, are critical determinants of stability under heat exposure.

InnoBoNT/A contains methionine and polysorbate 20, excipients known to mitigate oxidative and thermal degradation of proteins.^14^ Methionine can act as a sacrificial antioxidant, scavenging reactive oxygen species and preventing oxidative damage, whereas polysorbates stabilize proteins against aggregation and denaturation under agitation and elevated temperatures.^23-28^ This may explain the superior thermal resistance observed in the BoNT/A liquid formulation over powder-based BoNT/A products.

The clinical relevance of these results is underscored by the increasing popularity of EBDs for facial rejuvenation and skin tightening. Although most EBD devices are designed to raise tissue temperatures to ∼40°C to 80°C, increasing the frequency (Hz) to enhance accumulated energy can lead to a continuous rise in subdermal tissue temperature. Even with epidermal cooling techniques, residual thermal elevation in the dermis may persist. In the case of HIFU, the focused “hot ball” region is known to raise local tissue temperatures to over 60°C, depending on energy levels, thereby inducing collagen remodeling through tissue denaturation.^29^ Similarly, Sofwave technology using SUPERB has been reported to elevate tissue temperature in a cylindrical distribution.^30,31^ Monopolar RF treatment also raises tissue temperature to target levels through continuous, repetitive irradiation.^32^ More recently, microwave-based facial treatments have also been reported to increase tissue temperature through accumulated energy.^33^

From a clinical perspective, several implications emerge. First, when feasible, EBD treatments should be performed before botulinum toxin injection to eliminate the risk of in situ thermal degradation of the toxin. Second, if EBD procedures must follow toxin injection, clinicians may consider selecting formulations with demonstrated thermal stability, such as liquid BoNT/A formulations, to minimize the potential risk of reduced efficacy. These considerations may help optimize outcomes when combining EBD modalities with botulinum toxin treatments in aesthetic practice.

This study, however, has several limitations. Interpretation of these findings should take into account the inherent variability of mouse LD_50_ bioassays. In a previous study, the authors have shown that both intra-laboratory and inter-laboratory variability can be substantial because of biological and procedural factors.^34^ Although identical internal procedures were applied across all formulations in this study, the findings should be interpreted within the context of this inherent assay variability. The experiments were not designed to compare the intrinsic thermal stability of marketed drug products, because powder formulations were evaluated after reconstitution in saline rather than in their lyophilized state. Thus, the findings primarily reflect the stability of reconstituted toxins under in vitro heat exposure. Furthermore, in vivo conditions after intramuscular injection may differ substantially from those in vitro, as tissue perfusion, extracellular matrix interactions, and local heat dissipation could influence toxin stability during EBD treatment. Lastly, although excipients such as methionine and polysorbate 20 appear to enhance stability, the precise mechanisms by which they mitigate heat-induced denaturation remain unclear and require further investigation.

## CONCLUSIONS

This study demonstrated that only the liquid-stabilized BoNT/A formulation preserved biological potency after exposure to 60°C for up to 25 min, whereas all lyophilized formulations showed substantial potency loss under the same conditions. These results confirm that formulation characteristics play a critical role in thermal stability. Further studies are warranted to elucidate the underlying stabilizing mechanisms and to determine whether these findings are consistent across additional thermal conditions and experimental models.
